# Transcriptome-Wide Gene Expression Profiles from FFPE Materials Based on a Nuclease Protection Assay Reveals Significantly Different Patterns between Synovial Sarcomas and Morphologic Mimickers

**DOI:** 10.3390/cancers14194737

**Published:** 2022-09-28

**Authors:** Sabrina Borchert, Thomas Herold, Stavros Kalbourtzis, Rainer Hamacher, Yvonne Krause, Sophia Berger, Wiebke K. Guder, Arne Streitbuerger, Jendrik Hardes, Moritz Goetz, Sebastian Bauer, Hans-Ulrich Schildhaus

**Affiliations:** 1 Institute of Pathology, University Hospital Essen, Hufelandstraße 55, D-45147 Essen, Germany; 2 German Cancer Consortium (DKTK), Partner Site University Hospital Essen, D-45147 Essen, Germany; 3 Department of Medical Oncology, Sarcoma Center, West German Cancer Center, University Hospital Essen, D-45147 Essen, Germany; 4 Department of Orthopedics and Tumor Orthopedics, University Hospital Muenster, 48149 Muenster, Germany; 5 Department of Orthopedic Oncology, University Hospital Essen, D-45147 Essen, Germany; 6 Department of Musculoskeletal Oncology, University Hospital Essen, D-45147 Essen, Germany; 7 Center of Pathology, Cytology and Molecular Pathology, D-41462 Neuss, Germany

**Keywords:** transcriptome profiling, nuclease protection assay, biomarker, FFPE, HTG EdgeSeq, extraction-free RNA workflow

## Abstract

**Simple Summary:**

The following study aimed to validate and test the feasibility, reliability, technical applicability robustness, and reliability of a new commercially available transcriptome profiling assay, being performed on formalin-fixed, paraffin-embedded (FFPE) patient samples. The patients were suffering from either synovial sarcoma or spindle cell sarcoma, which are morphologically similar tumors, but differ in molecular characteristics. Transcriptome analysis of FFPE material is still challenging. However, it is the most available material in pathological routine and therefore valuable for translational research approaches. This new commercially available assay is based on a nuclease protection assay and has shown to be a feasible method for adequate transcriptome profiling with low sample input and therefore is suitable for further research of biomarkers.

**Abstract:**

Background: Transcriptome profiling provides large data on tumor biology, which is particularly valuable in translational research and is becoming more and more important for clinical decision-making as well. RNA sequencing is considered to be the gold standard for this. However, FFPE material, as the most available material in routine pathology, has been an undefeatable obstacle for RNAseq. Extraction-free nuclease protection assays have the potential to be a reliable alternative method for large-scale expression profiling. The aim of this study was to validate and test the basic feasibility, technical applicability robustness, and reliability of the HTG transcriptome profiling (HTP) assay on clinical tumor samples. Methods: FFPE samples from 44 synovial sarcomas (SyS) and 20 spindle cell sarcomas (SpcS) were used. The HTP assay was performed on 10 µm thin FFPE slides. After nuclease protection in the HTG Edge Seq System, libraries were generated for sequencing on an Illumina NextSeq 500 platform. Fastq data were parsed and then analyzed by using the HTG analysis platform EdgeSeq REVEAL. Immunohistochemistry was performed to validate the expression of TLE1. Results: The technical application of the HTP Panel revealed robust and reliable results with 62 samples, and only 2 samples failed due to an incomplete digestion of gDNA. The analysis, performed at the analysis platform REVEAL, showed 5964 genes being significantly differentially expressed between SpcS and SyS. In particular, overexpression of the known marker TLE1 in synovial sarcoma could be recovered, which underlines the reliability of this system. Discussion: Transcriptome profiling gets more and more important for tumor research and diagnostics. Among other established technologies, the HTP Panel has shown to be a feasible method to get robust and reliable results. Thereby, this method needs very few sample-input by getting a success-rate of 96.88%, which indicates the upper average range, compared to other technologies working with FFPE tissue. Conclusion: The nuclease protection assay-based HTP Panel is a feasible method for adequate transcriptome profiling with low sample input and therefore is suitable for further research of biomarkers.

## 1. Introduction

Clinical evaluation of tumors creates and uses primarily formalin-fixed and paraffin-embedded (FFPE) tissue [1]. Therefore, archives of FFPE tissue blocks represent an immeasurable resource for translational research. Analysis of molecular markers on FFPE materials, however, is challenging, since fixation treatment of the tissue causes degradation of nucleic acids [2] In particular, accurate transcriptome profiling on FFPE material has been an undefeatable obstacle for many years [3,4,5,6,7,8].

RNA sequencing (RNA-Seq) is the gold standard to measure whole transcriptomes of samples, but the reliability is limited with this method when FFPE samples are used. Recently, a new method for transcriptome analysis on FFPE tissue was established by HTG molecular, named the “HTG transcriptome profiling” (HTP). It is based on the Edge Seq System, which uses an extraction-free, nuclease protection assay. The analysis could be performed directly from FFPE slides in less than three days. Measuring 19,398 targets, this assay covers a large scale of relevant genes.

To test the robustness and reliability of this new method, we performed it on 64 patient samples, with 20 patients suffering from spindle cell sarcoma not otherwise specified (NOS) and 44 patients suffering from synovial sarcoma, which are characterized by the chromosomal translocation involving SS18 and either SSX1, SSX2, or rarely SSX4 [9]. These gene fusions orchestrate a specific transcriptional program, which determines morphologic appearance and biologic behavior of synovial sarcomas. There are, however, spindle cell sarcomas that share tumor morphology but behave clinically differently. Thus, we compared a genetically determined tumor entity with morphologic mimickers by applying a genome-wide transcriptional analysis.

## 2. Materials and Methods

### 2.1. Study Design

**Figure 1:**
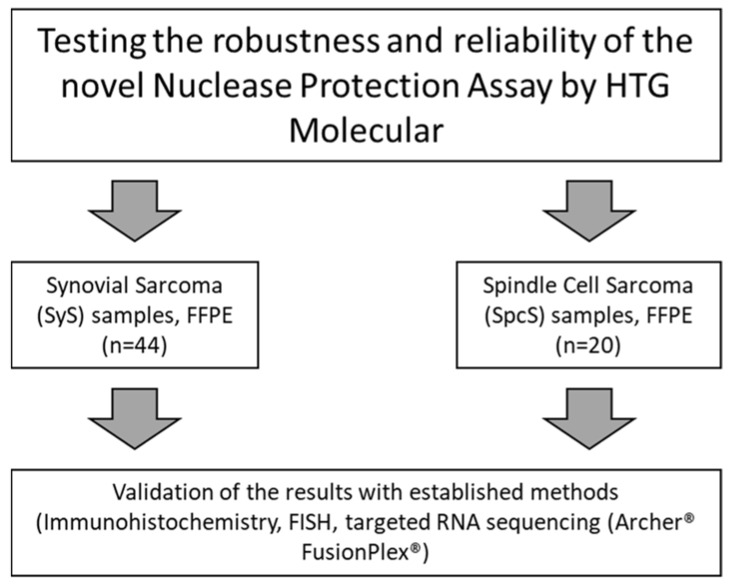
Study design. The robustness and reliability of the novel nuclease protection assay by HTG Molecular was tested on FFPE samples of 44 SyS and 20 SpcS patients. Samples were validated with results from established methods: immunohistochemistry, fluorescence in situ hybridization as well as targeted RNA sequencing (Archer^®^ FusionPlex^®^).

In this study, we aimed to test the robustness and reliability of the HTG transcriptome panel assay (HTG Molecular, Tucson, AZ, USA). Therefore, we used FFPE samples derived from 20 spindle cell sarcoma patients and 44 synovial sarcoma patients (see Figure 1). Patient samples exhibiting synovial sarcoma were pre-characterized by either Fluorescence-in-situ-hybridization (FISH) or the Archer^®^ FusionPlex^®^ Sarcoma panel (Invitae, San Francisco, CA, USA), a targeted sequencing method using the RNA of patient samples.

### 2.2. RNA-Isolation and Quantification for Archer^®^ FusionPlex^®^ Analysis

FFPE sections were stained with hematoxylin and eosin (H&E) and regions of interest were marked by two experienced pathologists for subsequent macrodissection. Macrodissection was performed on 2–6 10 µm thick FFPE sections and RNA was isolated by using the Maxwell^®^ RSC RNA FFPE Kit (AS1440, Promega, WI, USA), according to the manufacturer’s instructions. RNA was eluted in 50 µL RNase-free water and stored at −40 °C.

Measurement of RNA concentration was performed via fluorometric quantification (Qubit, Thermo Fisher Scientific, Waltham, MA, USA) using the RNA High sensitivity assay kit according to the manufacturer’s instructions. One µL of each isolated RNA sample was applied for measurement.

### 2.3. Archer^®^ FusionPlex^®^ Targeted RNA Sequencing

The detection of fusions in the tested samples was performed by using the Archer^®^ FusionPlex^®^ Sarcoma (ARSAR) Panel (Invitae, San Francisco, CA, USA). A total of 150 ng of each RNA sample were applied for cDNA synthesis. cDNA libraries were prepared via anchored multiplex PCR according to the manufacturers’ instructions (Archer^®^ FusionPlex^®^ Protocol for Illumina^®^, AB0005-8-reactions), leaving out the PreSeq RNA QC assay. The Archer^®^ FusionPlex^®^ Protocol for Illumina^®^ includes Archer^®^ Universal RNA Reagent Kit for Illumina^®^ (Catalog #SK-0093-8), Archer MBC adapters (Catalog #SA0043), and custom designed gene-specific primer (GSP) pool kit. The quality and quantity of cDNA libraries were assessed by using the D5000 Screen Tape assay on the Agilent 2200 TapeStation system (Santa Clara, CA, USA). Libraries were pooled (8–24 samples per run) and sequenced on an Illumina MiSeq^®^ system (SY-410-1003, Illumina, San Diego, CA, USA).

### 2.4. RNA-Fusion Detection via Archer^®^ FusionPlex^®^ Analysis Software

Fusion detection of raw sequence data was analyzed by using the Archer^®^ Analysis 6.2.1 User Software (Invitae, San Francisco, CA, USA). Fusions were verified bioinformatically by validation of the breakpoints.

### 2.5. Fluorescence In Situ Hybridization

Fluorescence in situ hybridization (FISH) was performed with formalin-fixed, paraffin-embedded, 1.5 µm thick sections. The tumor area was marked on the back of the slide by using a diamond pin. Sections were processed by using the VP2000 Processor (Abbott Laboratories, Chicago, IL, USA) as follows:

Day 1: Deparaffinization in xylene for 10 min for three times (3×), two times (2×) 5 min 100% ethanol, 96% ethanol (1 min), 80% ethanol (1 min), 70% ethanol (1 min). Slides were air-dried at 37 °C for 5 min and subsequently incubated in 0.2 M HCL (20 min), de-ionized (DI) water (3 min), 2× SSC wash buffer (3 min), pretreatment solution at 80 °C (30 min), DI water (1 min), 2× 5 min 2× SSC wash buffer, Protease I at 37 °C (90 min), 2× 5 min 2× SSC wash buffer, 4.5%-buffered formalin (10 min), 2× 5 min 2× SSC wash buffer, DI water (1 min), air-drying at 37 °C (5 min).

Subsequently, FISH-probe was applied on sections and covered with cover glasses. For detection of translocations involving the FUS gene, the ZytoLight^®^ 2C SPEC FUS Dual Color Break Apart Probe was used (ZytoVision GmbH, Bremerhaven, Germany, Z-2130-50). Fixogum (Marabu, Tamm, Germany) was used to seal the cover glass. Slides were placed in a hybridizer (Agilent Dako, Santa Clara, CA, USA). Denaturation was performed at 75 °C for 10 min followed by hybridization at 37° for 17–18 h.

Day 2: After hybridization, fixogum and cover glasses were removed, and post-hybridization wash was performed by using the VP2000 Processor: 2× SSC + 0.3% NP40 wash buffer at 72 °C (2 min), 2× SSC wash buffer at room temperature (1 min), DI water (1 min), 96% ethanol (1 min), air-drying (5 min).

DAPI counterstain was applied to dry specimens. Slides were covered with a cover glass and subsequently, signal enumeration was performed on the microscope (Leica DM6 B, Leica Microsystems CMS GmbH, Wetzlar, Germany.

### 2.6. Immunohistochemistry

Two selected samples of SpcS and SyS respectively, were stained immunohistochemically (IHC) against TLE1. For IHC staining, the deparaffinized and rehydrated tissue slides were incubated with the TLE1 antibody, (clone 1F5, monoclonal, diluted 1:50; CellMarque, Sigma-Aldrich, St. Louis, CA, USA). Subsequently, after antigen retrieval and washing steps, the tissue slides were incubated with a secondary antibody needed for DAB (3,3′-Diaminobenzidin) staining. TLE1 staining was evaluated by an experienced pathologist (HUS).

### 2.7. HTG Transcriptome Panel

Transcriptome analysis was performed by using the HTG Transcriptome Panel (HTG Molecular) according to the manufacturers’ instructions. Approximately 11 mm^2^ of tissue tumor area from each sample (FFPE slide of 10 µm thickness) were used to perform lysis reaction. Lysis reaction was performed by using the Lysis buffer A and included an additional DNase treatment step. After sample preparation, quantitative nuclease protection assay (qNPA) was performed on the HTG EdgeSeq Processor. In the next step, protection probes that hybridized to mRNA were amplified and tagged to generate sequencing libraries. After preparation of libraries, its concentration was determined by quantitative PCR using the Kapa Library Quantification Kit (Roche, Basel, Switzerland). Libraries were sequenced on an Illumina NextSeq 500 (Illumina, San Diego, CA, USA). Within 1 run, 24 sample libraries were sequenced by using the NextSeq 500/550 High Output Kit v2.5 (75 Cycles, Illumina, San Diego, CA, USA). Sequences were parsed directly from Fastq-files using the HTG EdgeSeq Parser software (HTG Molecular). Parsed data were analyzed via the Analysis platform Edge Seq REVEAL (HTG Molecular). Median normalization and subsequent differential expression (DE) analysis was performed. For DE analysis, the test method “DESeq2” was chosen. This differential expression analysis has been completed using the DESeq2 package (version 1.30.1, HTG molecular, Tucson, AZ, USA) available from Bioconductor. The DESeq2 package provides methods for estimating and testing differential expression using negative-binomial generalized linear models. Empirical Bayes methods are used to estimate dispersion and log2 (fold change) with data-driven prior distributions. See http://bioconductor.org/packages/release/bioc/vignettes/DESeq2/inst/doc/DESeq2.html (accessed on 26 April 2022) for more information.

No pre-filtering is applied to the data prior to analysis. The DESeq2 model corrects for library size using the median ratio method from Anders and Huber (2010). Dispersions are estimated with the Cox Reid-adjusted profile likelihood method developed by McCarthy et al. (2012). Log2 fold change is estimated via Tikhonov/ridge regularization with a zero-centered normal prior distribution with variance calculated using the observed distribution of maximum likelihood coefficients (see DESeq2 documentation for details). DESeq2 performs independent filtering on probes prior to applying the false discovery rate *p*-value adjustment in order to increase power. This will cause some probes to have no *p*-value.

## 3. Results

We analyzed the expression of around 20,000 gene targets in a cohort of 20 patients suffering from spindle cell sarcoma (SpcS) not otherwise specified (NOS) and 44 patients suffering from synovial sarcoma (SyS), exhibiting the classifying chromosomal translocation involving SS18 and either SSX1 or SSX2. The analysis was performed and visualized by using the Reveal software. The classifying chromosomal translocation involving SS18 and either SSX1 or SSX2 could also have been detected in each SyS sample. The samples have been pre-characterized by either FISH or targeted RNA Sequencing

The first step of the expression analysis was a look at the QC metrics. These are helpful to evaluate if the samples failed the QC. The software generates four plots: QC0, QC1, QC2, and QC3, which are explained in Table 1.

**Figure 2:**
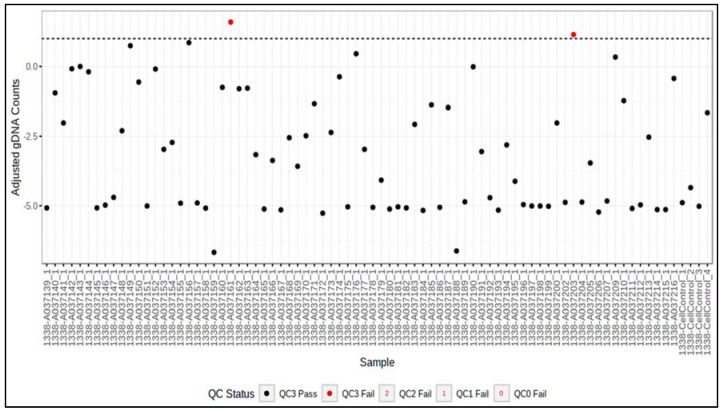
QC3-Plot of the HTP analysis. Two samples failed QC3 (red dots), which means that the digestion of gDNA did not complete, as they are above the dotted line meaning the threshold.

In our analysis, two samples failed the QC3, indicating an incomplete digestion of gDNA by DNase (Figure 2). These samples were excluded from further analysis.

**Figure 3:**
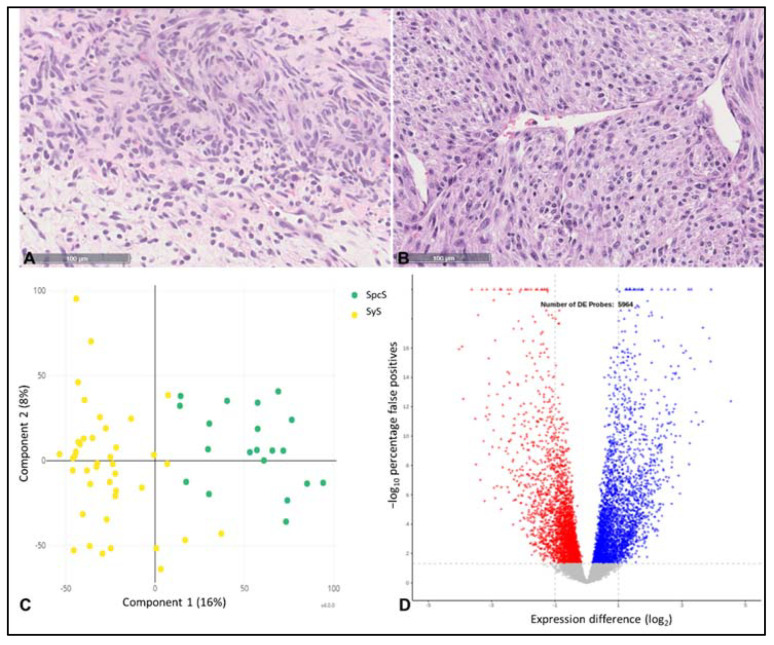
Differences between SyS and SpcS. (**A**) H&E staining of SyS. (**B**) H&E staining of SpcS. (**C**) This PCA plot depicts two distinct groups of expression pattern of genes within the two entities. (**D**) Volcano plot of tested genes. A total of 5964 genes showed differential expression when comparing synovial sarcoma vs. spindle cell sarcoma samples. Blue dots indicate elevated expression in synovial sarcomas and red dots depict elevated expression in spindle cell sarcomas. The *y*-axis indicates the log10 of the rank products percentage of false positives value as a function of the mean expression difference for the tested genes (adjusted *p*-value) and the *x*-axis indicates the log2 fold-change of gene expression.

SpcS and SyS are morphologically similar to each other, regarding the H&E staining (Figure 3A,B). However, the analysis of all tested samples revealed a distinct gene expression pattern, as shown in the principle component analysis (PCA) plot (Figure 3C).

The Volcano plot indicates 5964 genes being differently expressed with high significance (*p* < 0.001). This high number of genes showed distinct expression patterns in SpcS compared to SyS (Figure 3D).

**Figure 4:**
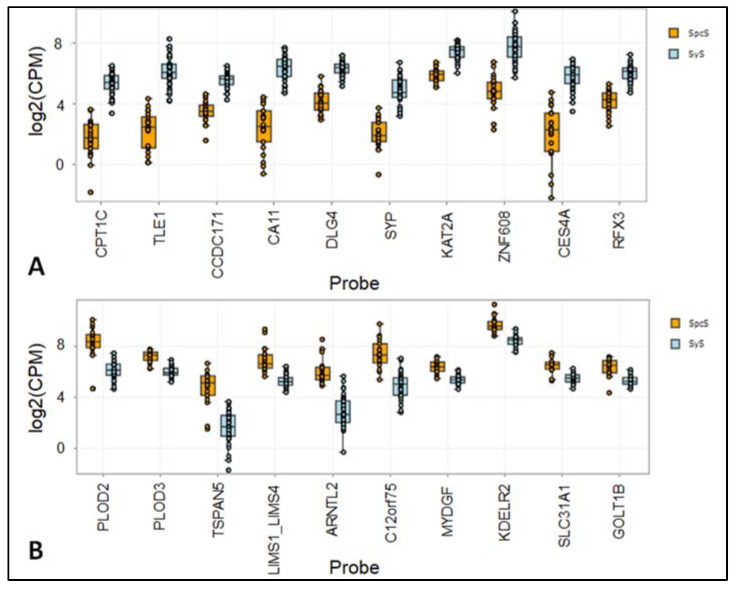
Differently expressed genes between SyS and SpcS. (**A**) Top 10 of the up-regulated genes in SyS and (**B**) top 10 of the up-regulated genes in SpcS are shown.

TLE1, CPTC1, CCDC171, CA11, DLG4, SYP, ZNF608, KAT2A, RFX3, and CES4A are upregulated in SyS. PLOD2, PLOD3, KDELR2, LIMS1:LIMS4, MYDGF, GOLT1B, TSPAN5, C12orf75, ARNTL2, and SLC31A1 are upregulated in SpcS. The top 10 down-regulated and up-regulated probes in SpcS and SyS, are shown in Figure 4A,B. All genes shown in Figure 4A,B showed *p*-values < 0.001. Table 2 indicates fold-change and FDR-adjusted *p*-values of the top 10 differentially expressed genes.

**Figure 5:**
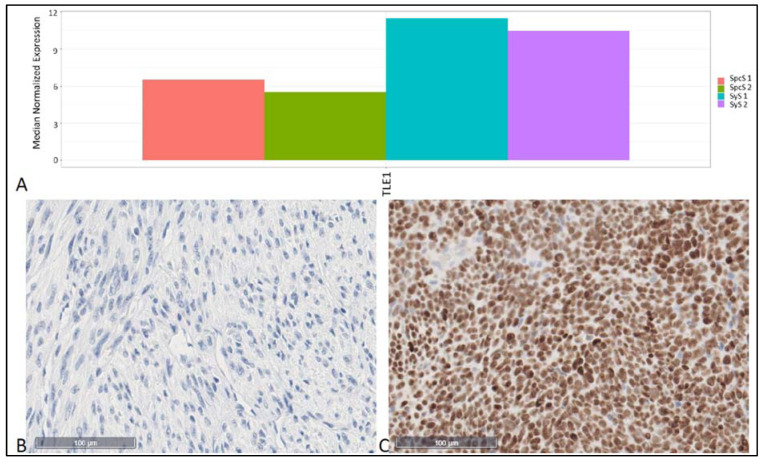
Different expression of TLE1 in SyS and SpcS samples. (**A**) This bar plot shows the expression (HTP analysis) of TLE in two samples of SyS and SpcS, respectively. TLE1 showed significantly higher expression in SyS (*p* = 6.14 × 10^43^). (**B**,**C**) IHC-staining of TLE1 in SpcS (**B**) and SyS (**C**).Magnification bars in B and C indicate 100 µm.

Especially expression of TLE1 showed highly significant differences (6.14 × 10^43^) between SyS and SpcS. Since TLE1 is a well-established marker for SyS, it is an interesting observation that this marker is one of the mostly significant genes. For this reason, we immunohistochemically stained SyS and SpcS against TLE1 (Figure 5B,C) and additionally generated a Bar plot, showing the expression of TLE1 in two samples of Sys and SpcS, respectively (Figure 5A).

**Figure 6:**
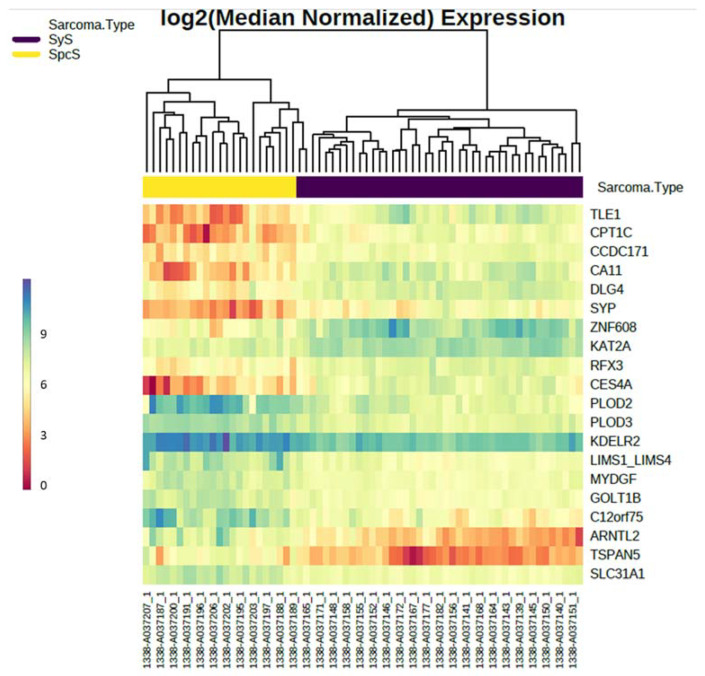
Heatmap of the top 20 differentially expressed genes. Blue bars specify Sys samples, yellow bars specify SpcS samples. The value of expression is colored from blue to red. High expression is indicated by blue (value = 10) and low expression by the red (value = 0) boxes. Yellow bars indicate a value of 6).

To visualize the top 10 differentially expressed genes in SpcS and SyS, a heatmap was created (Figure 6). Yellow bars indicate SpcS samples, and blue bars indicate SyS samples. Red designates low expression, whereas blue specifies an elevated expression of genes. This heatmap visualizes the clear differences in gene expression of the 10 genes, including the established marker TLE1 for SyS.

## 4. Discussion

Transcriptome profiling makes it possible to give an overview of gene expression in a large scale and thus simplifies the search for molecular biomarkers. For this purpose, RNA sequencing is considered the gold standard [10]. However, this method has its limitations using FFPE tissue. Nonetheless, FFPE tissue is the only available material, as it is routinely used and could be easily stored at room temperature. For this reason, sequencing methodologies using FFPE tissue are constantly under optimization to improve analysis on these challenging samples. We tested the HTP panel from HTG for sensitivity, robustness, and reliability of transcriptome-wide gene expression analysis on FFPE tissue.

### 4.1. Assay Performance

The extraction-free and nuclease-protection-based workflow simplified the practical handling. Additionally, with 11 mm^2^ of a 10 µm tissue slide, very few sample inputs are needed. This is especially important for samples where only biopsy material is available.

Our analyses revealed genes showing distinct expression patterns in SpcS compared to SyS. Regarding Figure 3C, the expression patterns of SyS compared to SpcS differ significantly and the volcano plot (Figure 3D) shows that 5964 genes are differentially expressed in these 2 entities. As we generally expected different gene expression patterns when comparing two different entities, these findings underline the robustness and reliability of the assay. In SyS, the most significantly result of differences in gene expression was shown for TLE1, being upregulated in SyS. This reflects the reliability of this assay, as TLE1 is an already established marker for SyS, being overexpressed in the nuclei of synovial sarcoma cells [11]. The immunohistochemical staining in Figure 5 is shown to validate the results gained by the novel nuclease-protection assay. As IHC is routinely performed in pathological diagnostic of SyS, we added this figure to underline the reliability of these results. In addition, a significantly different expression of other genes relevant for tumor progression could be found, e.g., Carnitine palmitoyltransferase 1 C (CPT1C), which promotes tumor growth and drug resistance, being reported for gastric cancer [12]. CPT1C was identified to be involved in fatty acid metabolism, due to its enzyme activity, which allows the entry of long-chain fatty acids [13]. Reilly and Mak reported unusual and enhanced expression of CPT1C in brain cancers and several sarcomas of soft-tissues and lung [14]. However, the detailed role or CPT1C in cancer remains indefinite [13]. Procollagen-lysine, 2-oxoglutarate 5-dioxygenase (PLOD) 3 was overexpressed in SpcS and is reported to promote tumor progression and poor prognosis in gliomas [15]. In addition, PLOD2 expression is elevated in SpcS. Qi and Xu reported PLODs to have enhancing effects of cell migration, invasion, and proliferation potentially by the modulation of collagen cross-link and maturation [16]. Our analyses have shown that the HTP panel is a suitable method to perform gene expression profiling on a large scale.

### 4.2. Comparison of HTP with Other Existing mRNA Profiling Systems

Currently, there are several established techniques including Lexogen QuantSeq, Qiagen QiaSeq, BioSpyder TempO-Seq, Ion AmpliSeq, Nanostring, Affymetrix Clariom S or U133A, Illumina BeadChip and RNA-seq enabling the analysis on FFPE or fresh frozen (FF) tissue [17]. Turnbull et al. compared these techniques in 2020 in terms of biochemistry, sample throughput, number of primers and mapped gene IDs (Ensembl), read depths, input of FFPE sample RNA, costs per sample, success rate of FF samples, and success rate of FFPE samples. To supplement this table with info about the HTP panel performed in this study, we listed these parameters in Table 3.

The HTP Panel of HTG Molecular has a moderate sample throughput (24 samples per 3 days), but the number of targets (19398) as well as the sequencing depth (8 M) is close to comparable methods such as BioSpyder TempO-Seq [17]. However, the costs per sample are very high in comparison to the other technologies. Nevertheless, its sensitivity and success rate of FFPE samples is the highest compared to others, considering the low RNA input needed for HTP. In addition, FFPE blocks used in this study were stored for up to five years. HTG Molecular itself reports robust results for FFPE material older than 10 years [10].

## 5. Conclusions

The HTG transcriptome profiling is a feasible method for gene expression analysis in a large scale. Our performed transcriptome profiling revealed robust results, and we were able to retrieve the known significant overexpression of TLE1 in synovial sarcoma. Therefore, this method of transcriptome profiling has proven to be a very robust method for the search of biomarkers, especially in limited tumor material. Reproducible results were achieved, while very small amounts of RNA are needed.

## Figures and Tables

**Table 1:** QC plots and their meaning.

QC Metric	Failure Mode Detected	Criteria
**QC0**	Insufficient RNA	%POS > 4% is a failure
**QC1**	Insufficient read depth	Total aligned reads < 7 million per sample is a failure
**QC2**	High background signal	Median log2 (CPM) negative control probes > 2 is a failure
**QC3**	Incomplete digestion of gDNA by DNase	Median adjusted log2 (CPM) of gDNA probes > 1 is a failure

**Table 2:** Data of the top 10 differentially expressed genes between SyS and SpcS. Mean normalized values for SyS and SpcS, average expression, fold change, raw *p*-value, and false-discovery-rate (FDR)-adjusted *p*-values are shown.

Probe	Mean Normalized SyS	Mean Normalized SpcS	AveExpr	Fold Change SpcS. Vs. SyS	rawP SpcS. Vs. SyS	adjP SpcS. Vs. SyS
CPT1C	769	87	9.07	−8.89	6.29 × 10^−48^	7.57 × 10^−44^
TLE1	1520	123	10.03	−12.38	1.02 × 10^−46^	6.14 × 10^−43^
CCDC171	870	242	9.36	−3.60	1.73 × 10^−43^	6.94 × 10^−40^
CA11	1593	162	10.11	−9.87	2.09 × 10^−42^	6.29 × 10^−39^
DLG4	1472	392	10.11	−3.75	5.39 × 10^−39^	1.30 × 10^−35^
PLOD2	1366	8094	11.83	5.93	3.08 × 10^−36^	6.17 × 10^−33^
PLOD3	1170	2982	10.80	2.55	1.49 × 10^−35^	2.56 × 10^−32^
SYP	611	94	8.77	−6.48	1.43 × 10^−34^	2.16 × 10^−31^
KAT2A	3135	1225	11.28	−2.56	1.39 × 10^−30^	1.51 × 10^−27^
ZNF608	4609	691	11.68	−6.67	1.50 × 10^−30^	1.51 × 10^−27^

**Table 3:** Table of technology parameters from the comparison of Turnbull et al. [17], supplemented with parameters of HTP performed in our study (marked in bold).

Technology	Technology /Platform	Biochemistry	Approx. Throughput	Max. no. probes/Primer Pairs	No. of Mapped ENSG IDs	Read Depths	Input FFPE RNA (ng)	Approx. Cost per Sample (£)	Success Rate of FFPE Samples (n)
3′ RNA sequencing	Lexogen QuantSeq	RNA → RT, oligodT priming from 3′ end, random priming towards 3′ end → amplification and barcoding → sequencing	96 samples per 5 days	55,765	25,610	10 M	500	90	98% (318)
	QiaSeq UPX 3′ Transcriptome	RNA → RT, oligodT priming for cDNA synthesis →template switching for 2nd strand synthesis priming → fragmentation → end repair addition, adapter ligation → PCR to add indices → sequencing	96 samples per 5 days	42,553	20,000	15 M	10	50	94% (48)
Specific Targeted Sequencing	BioSpyder TempO-Seq	RNA → annealed 50 bp detector oligos are ligated then amplified and barcoded → sequencing	192 samples per 4 days	19,300	19,300	12 M	20 μm FFPE Section	160	95% (38)
	Ion Ampliseq Transcriptome	RNA → RT, multiplex PCR → sequence barcoding → emulsion PCR → sequencing of ~150 bp Targets	96 samples per 5 days	20,802	19,059	8 M	10	160	76% (76)
	**HTG Edge Seq, Illumina Next Seq 500**	**FFPE slide → nuclease protection and probe hybridization → S1 nuclease digestion → barcoding and amplification → Library Cleanup → sequencing → Data Parsing**	**24 samples per 3 days**	**19,398**	**19,398**	**8 M**	**11 mm^2^** **on a 10 µm FFPE section**	1159,095	**96.88** **(64)**
Targeted Probes	Nanostring	RNA → hybridisation to fluorescent barcoded probes in solution → immobilised in nCounter cartridge → scan	12 samples per day (800 genes)	800	800	N/A	50	250	100% (12)
Newer Microarray	Affymetrix Clariom S	RNA → cRNA amplification → hybridisation to GeneChip → scan	192 samples per 4 days	211,300	>20,000	N/A	50	100	100% (8)
Traditional Microarray	Affymetrix U133A		192 per day	250,833	11,827	N/A	50	360	100% (286)
	Illumina BeadChip HT12 v3/v4	RNA → RT, amplification, biotinylation (NuGEN WT Ovation kit) → hybridisation to 50 bp probes on chip → scan	96 samples per 1.5 days	47,323	22,571	N/A	1500	195	21% (206)
Full RNA Sequencing	RNA-seq	RNA → fragmentation → RT → barcoded library construction → genome-wide full RNA sequencing	8 samples per 5 days	20,025	18,571	136 M paired reads	2000	250-500	100% (87)

## Data Availability

The data presented in this study are available on request from the corresponding author. The data are not publicly available due to privacy restrictions.
